# FgMon1, a guanine nucleotide exchange factor of FgRab7, is important for vacuole fusion, autophagy and plant infection in *Fusarium graminearum*

**DOI:** 10.1038/srep18101

**Published:** 2015-12-10

**Authors:** Ying Li, Bing Li, Luping Liu, Huaigu Chen, Haifeng Zhang, Xiaobo Zheng, Zhengguang Zhang

**Affiliations:** 1 Department of Plant Pathology, College of Plant Protection, Nanjing Agricultural University, and Key Laboratory of Integrated Management of Crop Diseases and Pests, Ministry of Education, Nanjing 210095, China; 2 Institute of Plant Protection, Jiangsu Academy of Agricultural Sciences, Nanjing 210014, China

## Abstract

The Ccz1-Mon1 protein complex, the guanine nucleotide exchange factor (GEF) of the late endosomal Rab7 homolog Ypt7, is required for the late step of multiple vacuole delivery pathways, such as cytoplasm-to-vacuole targeting (Cvt) pathway and autophagy processes. Here, we identified and characterized the yeast Mon1 homolog in *Fusarium graminearum*, named FgMon1. *FgMON1* encodes a trafficking protein and is well conserved in filamentous fungi. Targeted gene deletion showed that the ∆*Fgmon1* mutant was defective in vegetative growth, asexual/sexual development, conidial germination and morphology, plant infection and deoxynivalenol production. Cytological examination revealed that the *∆Fgmon1* mutant was also defective in vacuole fusion and autophagy, and delayed in endocytosis. Yeast two hybrid and *in vitro* GST-pull down assays approved that FgMon1 physically interacts with a Rab GTPase FgRab7 which is also important for the development, infection, membrane fusion and autophagy in *F. graminearum*. FgMon1 likely acts as a GEF of FgRab7 and constitutively activated FgRab7 was able to rescue the defects of the ∆*Fgmon1* mutant. In summary, our study provides evidences that FgMon1 and FgRab7 are critical components that modulate vesicle trafficking, endocytosis and autophagy, and thereby affect the development, plant infection and DON production of *F. graminearum*.

*Fusarium graminearum* (teleomorph: *Gibberella zeae*) is an economically important plant pathogen that causes Fusarium head blight (FHB) or head scab disease on wheat and barley worldwide^1^,^2^,^3^. *F. graminearum* rapidly spreads during the heading-to-flowering stages when weather conditions are favorably wet^1^, and leads symptoms of premature bleaching as well as damage in grain yield. In addition to the high economic impact of FHB, the infected cereals are often contaminated with deoxynivalenol (DON) and zearalenones (ZEA) which poses an extremely threat to human and animal health^1^,^4^. However, efficient strategies to control the FHB have not been well established to date, and the current means is primarily dependent on fungicides that often exhibit many negative traits^5^,^6^. Therefore, it is of high urgency to identify the molecular mechanism of *F. graminearum* on growth and disease, in order to develop novel and effective control strategies for FHB.

Mon1 and Ccz1 were the first identified genes which are essential for the cytoplasm to vacuole targeting (Cvt) pathway and autophagy in yeast^7^. Further evidences showed that Ccz1 and Mon1 are also essential for yeast homotypic vacuole fusion and regulating vesicle traffic at the tethering/docking stage^8^. Within the endomembrane system of eukaryotic cells, protein and lipid are packaged into vesicles at donor organelles and transported to acceptor membranes which depend on multiple fusion and fission events^9^,^10^. Soluble N-ethylmaleimide-sensitive factor attachment protein receptor (SNARE) proteins play an important role in intracellular membrane fusion in eukaryotic cells^11^. According to the “zippering model,” t-SNAREs exist in target membranes, while v-SNAREs are on the membrane of vesicles. Both can assemble into a trans-SNARE structure, forming a tight connection between the membranes and mediate the mixing of the lipid bilayers and promote fusion. After fusion, SNAREs convert the “trans” into “cis” construction^12^. Previous studies in yeast indicated that Ccz1 and Mon1 proteins were assembled into the end product of fusion, forming the cis-SNARE complex which directly participates in fusion^8^. In our previous studies, FgVam7, one of the SNAREs in *F. graminearum* was found to play a critical role in hyphal growth, conidial formation, plant infection and DON production. The *FgVAM7* gene deletion mutant exhibits a defect in vacuolar morphology and delayed endocytosis^13^.

In the endomembrane trafficking system, a conserved machinery is required that consist of Rab GTPases, tethering factors and the SNARE proteins^14^. Rab proteins can exist in both the active GTP- and inactive GDP-bound form. With the presence of guanine nucleotide exchange factors (GEFs), Rab proteins can be converted into their active GTP form thus to bind multiple effectors such as tethering factors and SNAREs to promote membrane fusion^15^,^16^,^17^,^18^,^19^,^20^. Once the Rab proteins exert its function, GTPase-activating protein (GAP) enhances the hydrolysis GTP to GDP, and thereby inactivating Rab^21^. In *Saccharamyses cerevisiae*, the Rab7 homolog Ypt7 was found to be localized mainly in the vacuolar membrane and is required for the vesicle docking and vacuole-to-vacuole fusion^22^. Recent studies in *F. graminearum* and *Magnaporthe oryzae* revealed that both FgRab7 and MoRab7 localize to the vacuolar membrane and regulate the fusion of vacuoles and autophagosomes^23^,^24^. Many recent studies have demonstrated that the Mon1-Ccz1 complex was the GEF for Ypt7 protein in yeast^25^. Deletion of either *MON1* or *CCZ1* in yeast leads to vacuole fragmentation^8^, the same as *YPT7* deletion mutant^26^. Besides, the *MON1* gene was identified in a knockout mutant that cause hypersensitive to brefeldin A and monensin that interfere with intracellular protein transport processes^27^. Furthermore, the Mon1-Ccz1 complex were also found to be essential for autophagy pathways^7^. For example, in *M. oryzae*, yeast Mon1 homolog MoMon1 was essential for fungal development, pathogenicity, vacuolar assembly and autophagy^28^.

Although the biological functions of Mon1 and Rab7/Ypt7 have been investigated in yeast, plants and *M. oryzae*, their roles and relations have not yet been identified in *F. graminearum*. Here, we characterized FgMon1 and FgRab7, and found that FgMon1 likely is a GEF of FgRab7 and directly interacts with FgRab7, FgRab7^Q67L^ (GTP-associated version) and FgRab7^T22N^ (GDP-associated version). Both FgMon1 and FgRab7 play critical roles in endocytosis, vacuole fusion and autophagy, thereby controlling the growth, asexual/sexual development, plant infection and DON production in *F. graminearum*.

## Results

### FgMon1 encodes a trafficking protein in *F. graminearum*

FgMon1 was identified via searching in the *F. graminearum* genome database with the yeast Mon1 protein as a query. *FgMON1* encodes a 601 amino acid (aa) protein with a well conserved trafficking protein domain Mon1 from resides 170 to 601. Phylogenetic analysis revealed that FgMon1 is also well conserved among different fungi (Figure S1). It shares a high amino acid sequence identity to its homologs from other fungi, with 92% aa identity to that in *Fusarium oxysporum*, 91% to *Fusarium verticillioides*, 76% to *Acremonium chrysogenum*, 75% to *Trichoderma harzianum*, 67% to *Ustilaginoidea virens*, 70% to *Metarhizium album*, 71% to *Colletotrichum gloeosporioides*, 70% to *Verticillium dahlia*, 67% to *M. oryzae*, 63% to *Neurospora crassa*, 33% to *Cryptococcus neoformans*, 31% to *S. cerevisiae* and 28% to *Candida albicans*, respectively.

### FgMon1 plays a critical role in growth and conidiogenesis

**Figure 1:**
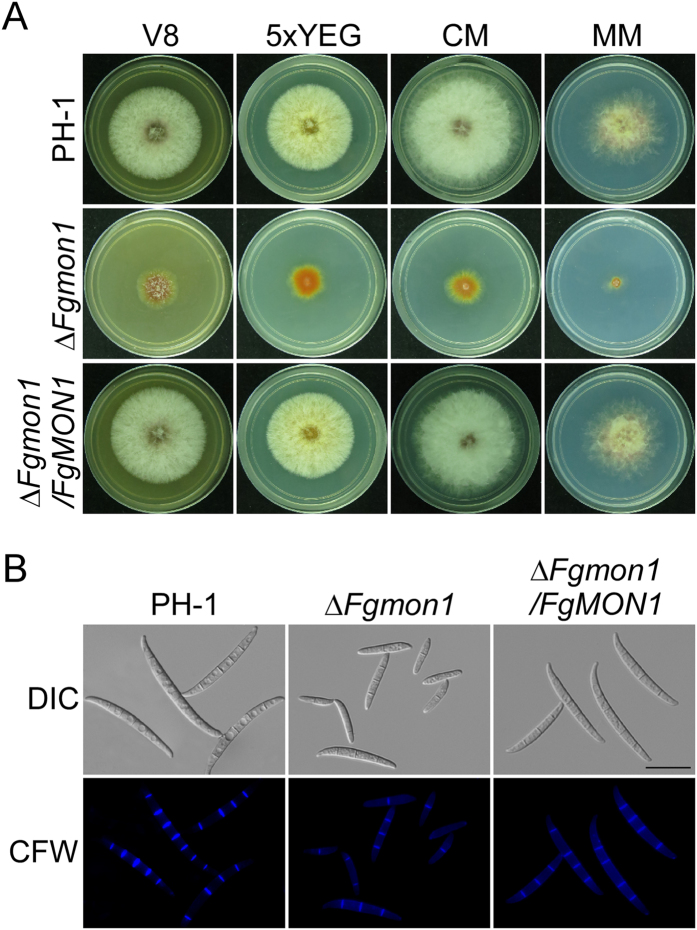
Colony morphology, vegetative growth and conidiogenesis defects of the ∆*Fgmon1* mutant. (**A**) Three-day-old cultures of the wild type PH-1, ∆*Fgmon1* mutant and the complemented transformant ∆*Fgmon1*/*FgMON1* on V8, 5xYEG, CM and MM plates. (**B**) Conidia of PH-1, ∆*Fgmon1* mutant and ∆*Fgmon1*/*FgMON1* were stained with Calcofluor white and examined by DIC or epifluorescence microscopy.

To investigate the roles of FgMon1 in *F. graminearum*, the *FgMON1* gene replacement construct was generated by split marker approach (Figure S2A) and transformed into the protoplast of wild type strain PH-1 as previously described^29^. The resulting transformants were screened by PCR and further confirmed by Southern blot analysis (Figure S2B). We first checked the growth and colony morphology of the *∆Fgmon1* mutant. Compared to the wild type PH-1 and complemented transformant *∆Fgmon/FgMON1*, the *∆Fgmon1* mutant showed less aerial hyphae and significantly reduced growth rate on V8, 5xYEG, CM and MM agar plates (Fig. 1A, Table 1). Conidial production of the *∆Fgmon1* mutant was quantified in CMC media and the number of conidia in the mutant was decreased to 17% of the wild type PH-1 (Table 1). Microscopy observation revealed that the *∆Fgmon1* mutant was also defective in conidial morphology. Conidia of the *∆Fgmon1* mutant were shorter and had fewer septa in comparison to the wild type and the complemented transformant. More than 50% conidia of the *∆Fgmon1* mutant have only one or two septa, while 91% conidia of PH-1 have three septa or more. The average conidial length of the *∆Fgmon1* mutant was 69% of that the wild type (Fig. 1B, Table 1). These results indicated that FgMon1 is important for vegetative growth, conidiation and conidial morphology.

### FgMon1 is involved in conidial germination and is essential for sexual reproduction

**Figure 2:**
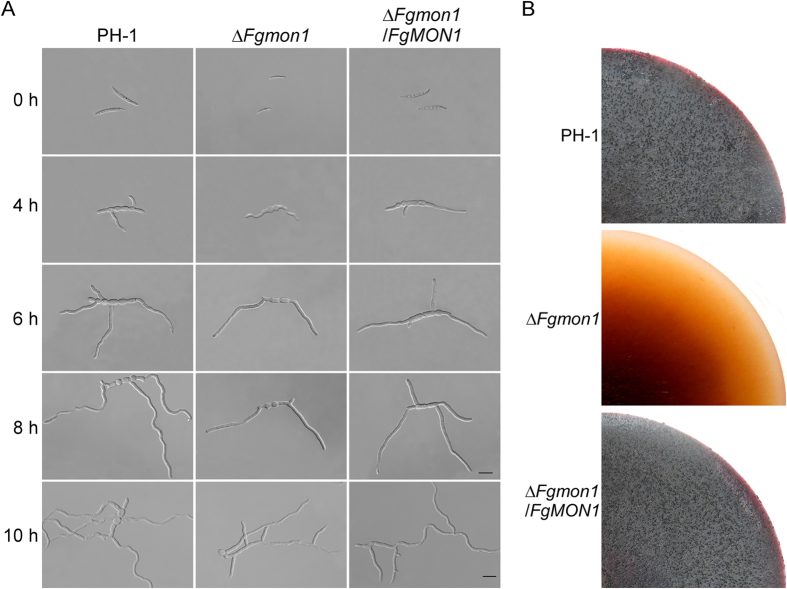
Assays for defects of the ∆*Fgmon1* mutant in conidial germination and sexual reproduction. (**A**) Conidia of PH-1, ∆*Fgmon1* mutant and ∆*Fgmon1*/*FgMON1* were incubated in liquid YEPD for 4, 6, 8 and 10 h and examined for germination and germ tube growth. Scale bar = 20 μm. (**B**) Self-crossing plates of the indicated strains at 10 days post-fertilization.

To examine whether FgMon1 has a role in conidial germination, we examined the conidial germination in liquid YEPD media. The result showed that conidia of the *∆Fgmon1* mutant were able to germinate from the end cells but delayed to germinate from middle cells (Fig. 2A). Even when incubation for 16 h, multipolar germination rate of the mutant conidia was remain significantly lower than that of the wild type (Table 2). After 18 h incubation, the mutant and wild type showed a similar multipolar germination rate (Table 2). Because ascospores play a crucial role in the disease cycle of *F. graminearum*, we also assayed sexual reproduction of PH-1, *∆Fgmon1* mutant and *∆Fgmon/FgMON1* on carrot agar plates as previously described^13^. After 10 days of inoculation, the PH-1 and the complemented tranformant produced numerous mature perithecia. In contrast, the *∆Fgmon1* mutant failed to produce perithecia under the same conditions (Fig. 2B). These results suggested that FgMon1 is involved in conidial germination and plays an essential role in sexual reproduction in *F. graminearum*.

### FgMon1 is important for plant infection and DON production

**Figure 3:**
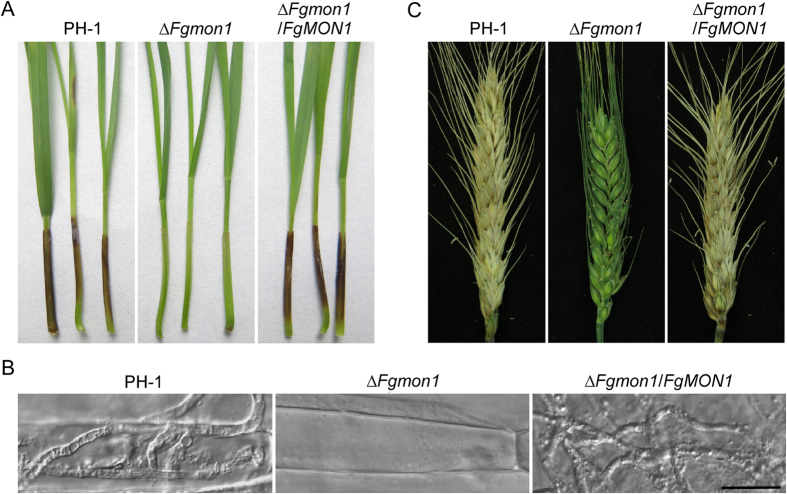
Defects of the ∆*Fgmon1* mutant in plant infection. (**A**) Wheat germ were inoculated with conidial suspensions and examined at 10 days post inoculation (dpi). (**B**) Examination the infectious hyphae of the indicated strains on wheat coleoptiles at 2 dpi. Scale bar = 20 μm. (**C**) Flowering wheat heads were inoculated with conidia of PH-1, ∆*Fgmon1* mutant and ∆*Fgmon1*/*FgMON1*, and photographed at 14 dpi.

To determine the role of FgMon1 in plant infection, we first assayed the *∆Fgmon1* mutant on wheat germs by droplet inoculation. After incubation at 25 °C for 10 days, severe disease symptoms were observed on the wheat coleoptiles inoculated with conidia suspensions prepared from wild type and complemented transformant. In contrast, the *∆Fgmon1* mutant almost caused no symptoms on the wheat coleoptiles under the same conditions (Fig. 3A). Microscopy examination revealed that the mutant was unable to penetrate through the wheat coleoptile epidermis and no infectious hyphae were observed in plant cells, while the wild type and complemented transformant formed branching and expanded infectious hyphae in the cells (Fig. 3B). We further point-inoculated the flowering wheat heads with conidial suspensions. The *∆Fgmon1* mutant was also defective in plant infection. Wheat kernels nearby the inoculation sites remained healthy 14 days following inoculation, while all wheat kernels in the inoculated spikelets were infected by the wild type and complemented transformant (Fig. 3C). These results indicated that FgMon1 plays a critical role in plant infection in *F. graminearum*. Because DON was known as an important virulence factor in *F. graminearum*^30^, DON production was measured in the wheat kernels infected by PH-1 and the *∆Fgmon1* mutant. DON production was at a very low level in the *∆Fgmon1* mutant, only 0.016 mg of DON was detected in per milligram of ergosterol, while over 4 mg of DON in per milligram of ergosterol was detected in the wild type PH-1 (Table 1), suggesting FgMon1 has a critical role in DON production. We further test the expression level of trichothecene synthase genes *TRI5* and *TRI6* that are involved in DON biosynthesis. qRT-PCR analysis revealed that the expression of *TRI5* (decreased to 60% of the wild type) and *TRI6* (decreased to 50% of the wild type) was significantly decreased in the *∆Fgmon1* mutant. These results indicate that FgMon1 modulates DON biosynthesis by regulating the expression of *TRI5* and *TRI6* in *F. graminearum*.

### Expression and intracellular localization of GFP-FgMon1 in *F. graminearum*

**Figure 4:**
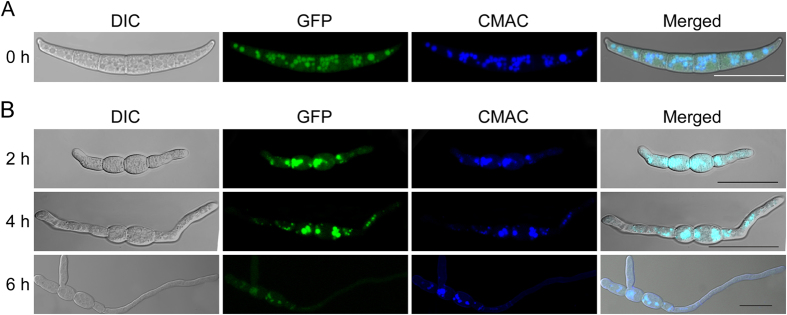
Expression and cellular localization of GFP-FgMon1 in conidia and germ tubes. Conidia (**A**) and germ tubes (**B**) expressing the GFP-*FgMON1* fusion construct or stained with CMAC were examined by DIC or epifluorescence microscopy. Scale bar = 20 μm.

To examine the expression and localization pattern of the GFP-FgMon1 proteins in *F. graminearum*, a GFP-*FgMON1* fusion construct was generated and transformed into the *∆Fgmon1* mutant. The conidia and germ tubes from the resulting transformant were observed under a fluorescence microscopy. Strong GFP signals were present mainly in the punctate structures of the cytosol, and weak fluorescence was observed in cytosol in both conidia and germ tubes. We further stained the conidia and germ tubes with CMAC (7-amino-4-chloromethylcoumarin), a dye that labels the lumen of fungal vacuoles. The results showed that CMAC exactly stained the above punctate structures, indicating GFP-FgMon1 localized in the vacuoles in *F. graminearum* (Fig. 4A,B).

### FgMon1 is involved in endocytosis and vacuole fusion

**Figure 5:**
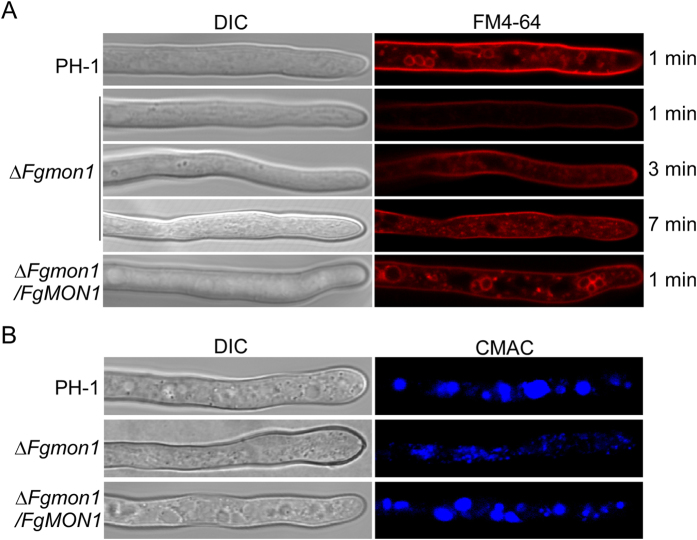
Observation of the defects of the ∆*Fgmon1* mutant in endocytosis and vacuole fusion. (**A**) Hyphae of PH-1, ∆*Fgmon1* mutant and ∆*Fgmon1*/*FgMON1* were stained with FM4-64 and examined by DIC or epifluorescence microscopy. (**B**) Hyphae of the indicated strains were stained with CMAC and examined by DIC or epifluorescence microscopy.

Because yeast Mon1 protein plays a critical role in cytoplasm-to-vacuole targeting (CVT) pathway^7^, we examined the endocytosis in the *∆Fgmon1* mutant by FM4-64 staining. The wild type PH-1 and the complemented transformant uptake FM4-64 dye within 1 minute (min) of exposure, and numerous intact endosomes were stained and observed in the hyphae. In contrast, no uptake occurred within 1 min in the *∆Fgmon1* mutant, while weak and normal uptake was only examined after 3 min or 7 min of exposure (Fig. 5A), suggesting that FM4-64 uptake was delayed in the mutant. Besides, many fragmentized structures replace the intact endosomes in the hyphae (Fig. 5A). We further examined the vacuoles of the *∆Fgmon1* mutant by CMAC staining, a dye labels the lumen of fungal vacuoles. Fragmentized vacuoles were observed in the mutant compared to normal intact vacuoles in the wild type and complemented transformant (Fig. 5B). These results indicated that FgMon1 plays a critical role in endocytosis and vacuole fusion.

### FgMon1 is indispensable for autophagy in *F. graminearum*

**Figure 6:**
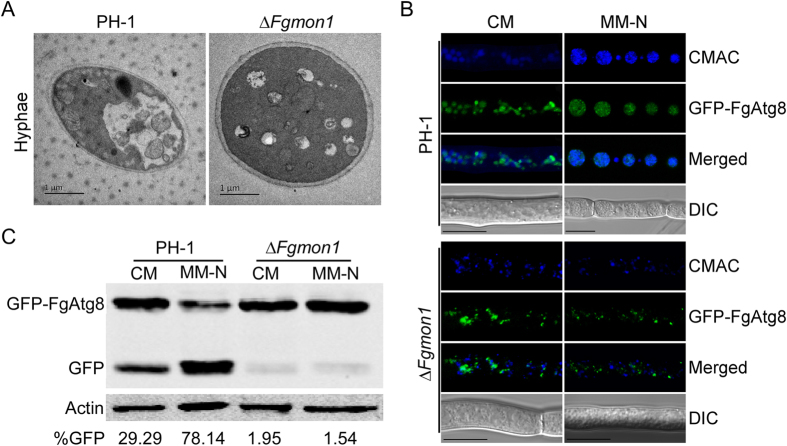
Assays for the defects of the ∆*Fgmon1* mutant in autophagy. (**A**) Organelles and autophagic bodies were observed in vacuoles under starvation conditions. (**B**) PH-1 and ∆*Fgmon1* mutant expressing GFP-FgAtg8 were grown in liquid CM medium at 25 °C for 10 h, and shifted to liquid MM-N medium with 2 mM PMSF for 8 h. Mycelia were stained with CMAC and examined by DIC or epifluorescence microscopy. Scale bar = 10 μm. (**C**) GFP-FgAtg8 proteolysis assays of PH-1 and ∆*Fgmon1* mutant. Mycelia cultured at 25 °C for 10 h in CM liquid medium were continuously shaken at 150 rpm. Autophagy was induced after 8 h of nitrogen starvation. Mycelia were collected and mycelia extracts were analyzed by western blot using anti-GFP.

The autophagy process is regulated by multiple autophagy-related proteins. Since the autophagy process also includes membrane trafficking and fusion events, proteins involved in vesicle trafficking such as SNAREs and Rab GTPases have been reported to be essential in autophagy^23^,^24^. Because the *∆Fgmon1* mutant was defective in endocytosis and vacuole fusion, we suppose that the mutant might also have defect in autophagy pathway. To test this possibility, vacuoles of hyphal cells were examined with starvation induction assays. We first examined the autophagic bodies under transmission electron microscopy. After cultured in liquid MM-N medium with 2 mM PMSF for 4 h, no autophagic bodies in the vacuole of the *∆Fgmon1* mutant was observed. However, numerous autophagic bodies were observed in the vacuole of wild type PH-1 (Fig. 6A). The autophagic process could be tracing-observed by monitoring the vacuolar delivery and breakdown of GFP-Atg8^31^. Under non-induction conditions (CM medium) for 10 h, GFP-FgAtg8 was localized in the punctuate structures in both wild type and the *∆Fgmon1* mutant, while in wide-type some of the punctuate structures are delivered to the vacuole for degradation. When induced under nitrogen starvation (MM-N medium) condition in the presence of 2 mM PMSF for another 8 h, GFP-FgAtg8 accumulated in the vacuoles of the wild type. However, GFP signals remain exist in the punctuate structures of the mutant but not in the surrounding CMAC stained fragmented vacuoles (Fig. 6B). We concluded that the fusion of autophagosomes and vacuoles was impaired in the Δ*Fgmon1* mutant. To further explain this observation, GFP-FgAtg8 proteolysis assay was performed. Under normal conditions, a clear full-length GFP-FgAtg8 band (40 kDa) and a slightly weak GFP band (26 kDa) was detected in the wild type with an anti-GFP antibody (Fig. 6C). When hyphae were shifted to MM-N conditions, a relatively weak full-length GFP-FgAtg8 band but a very clear GFP band was detected in the wild type. In comparison, a clear full-length band and a hardly-detected GFP band were detected in the *∆Fgmon1* mutant regardless of cultural conditions (Fig. 6C). These results implicated the *∆Fgmon1* mutant was defective in autophagy.

### FgMon1 plays an important role in response to vesicular transport inhibitor and cell wall perturbing agents

**Figure 7:**
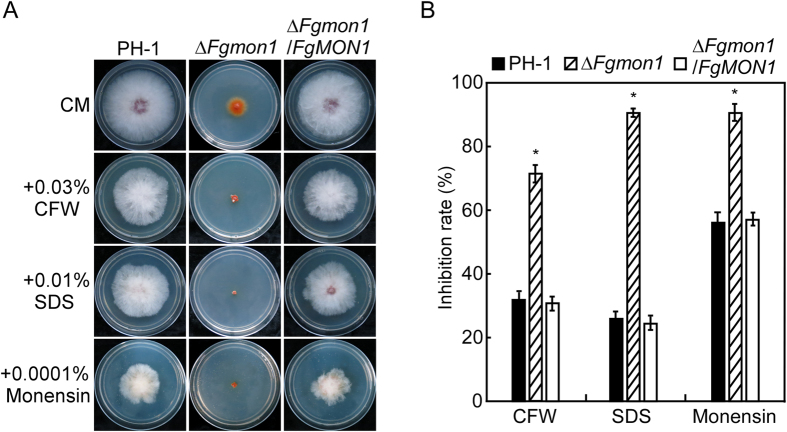
Defects of the ∆*Fgmon1* mutant in response to vesicular transport inhibitor and cell wall perturbing agents. (**A**) The wild type PH-1, ∆*Fgmon1* mutant and complemented transformant were inoculated on CM plates with CFW, SDS and monensin at 25 °C for 3 days. (**B**) Statistical analysis of the growth inhibition rate of the ∆*Fgmon1* mutant by different stressors in comparison to the wild type.

Because the *∆Fgmon1* mutant was defective in vacuole fusion, we speculated that this defect might affect the vesicular transport pathway and cell wall integrity. Therefore, the wild type PH-1, *∆Fgmon1* mutant and complemented transformant were inoculated onto the CM plates with cell wall perturbing agents (0.03% CFW, 0.01% SDS) and the drug that interferes with intracellular protein transport processes (0.0001% monensin). After 3 days incubation, the *∆Fgmon1* mutant showed an extremely small colony in comparison to that of the wild type on the plates (Fig. 7A). The growth inhibition rate of the mutant was increased 2-, 3- and 1.5-fold on CFW, SDS and monensin plates, respectively (Fig. 7B), indicating the *∆Fgmon1* mutant was hypersensitive to vesicular transport inhibitor and cell wall perturbing agents. It also implicates that FgMon1 had a role in vesicular transport pathway as well as maintenance of the cell wall integrity in *F. graminearum*.

### Constitutively activate FgRab7 could rescue the defects of the *∆Fgmon1* mutant

**Figure 8:**
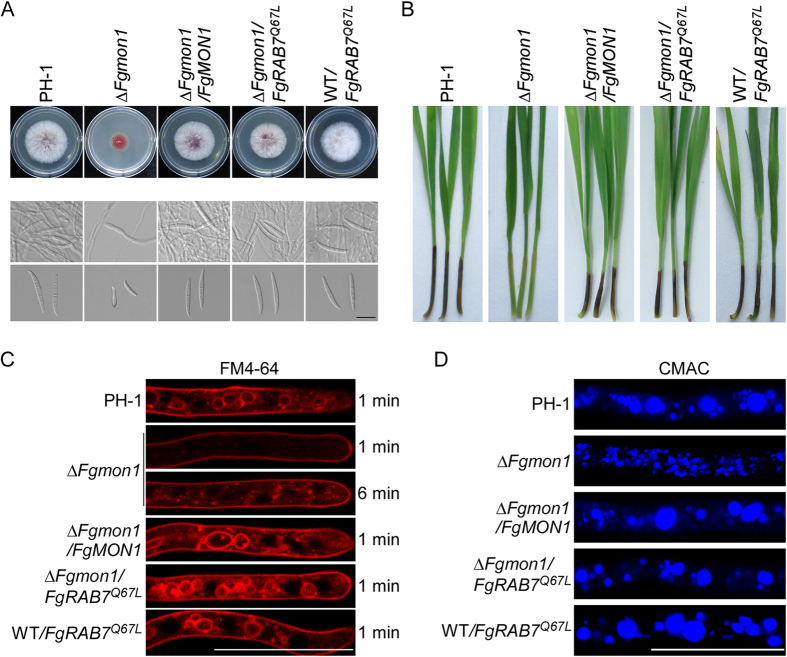
Constitutively activate FgRab7 could rescue the defects of the ∆*Fgmon1* mutant. (**A**) Vegetative growth, conidiation and conidial morphology of the wild type PH-1, ∆*Fgmon1* mutant, complemented transformant ∆*Fgmon1/FgMON1* and FgRab7 constitutively activate transformant ∆*Fgmon1/FgRAB7*^*Q67L*^ and WT/*FgRAB7*^Q67L^. (**B**) Pathogenicity of the indicated strains on wheat coleoptiles. (**C**) Endocytosis of the indicated strains evaluated by FM4-64 staining. (**D**) Vacuole morphology observation stained by CMAC. Scale bar = 20 μm.

Mon1-Ccz1 complex was known as the GEF of Rab7 homolog Ypt7 in yeast^25^. A latest study reported that Rab GTPase FgRab7 was essential for membrane trafficking-dependent growth and plant infection in *F. graminearum*^23^. Our independent work also showed that FgRab7 was important for the development of infection related morphogenesis, vacuole fusion and autophagy that is similar to the biological functions of FgMon1 (Figures S3 and S4). To figure out whether FgMon1 was a GEF of FgRab7 in *F. graminearum*, a construct encoding a constitutively activated FgRab7^Q67L^ (GTP hydrolysis defective) was transformed into the protoplast of the *∆Fgmon1* mutant. The resulting transformant *∆Fgmon1/FgRAB7*^Q67L^ was confirmed by qRT-PCR and showed 3.2-fold increased expression of *FgRAB7* compared to the wild type PH-1. Phenotype analysis revealed that the *∆Fgmon1/FgRAB7*^Q67L^ transformant displayed normal vegetative growth, conidiation, conidial morphology and virulence as the wild type PH-1 (Fig. 8A,B). Furthermore, the *∆Fgmon1/FgRAB7*^Q67L^ transformant also showed normal endocytosis and vacuole fusion by cytological examination (Fig. 8C,D). These results indicated that constitutively activated FgRab7 could rescue the defects of the *∆Fgmon1* mutant. In addition, we also transformed pYF11-*FgRAB7*^*Q67L*^ into the protoplast of wide-type PH-1. The resulting transformants WT/*FgRAB7*^Q67L^ were confirmed by qPCR, and showed a 3.3-fold increase of *FgRAB7* expression compared with the wild-type. Phenotype analysis revealed that WT/*FgRAB7*^Q67L^ showed no obvious changes on vegetative growth, conidiation, conidial morphology, pathogenicity as well as vacuole morphology and endocytosis (Table 1, Fig. 8A–D).

### FgMon1 physically interacts with FgRab7, FgRab7^Q67L^ and FgRab7^T22N^

**Figure 9:**
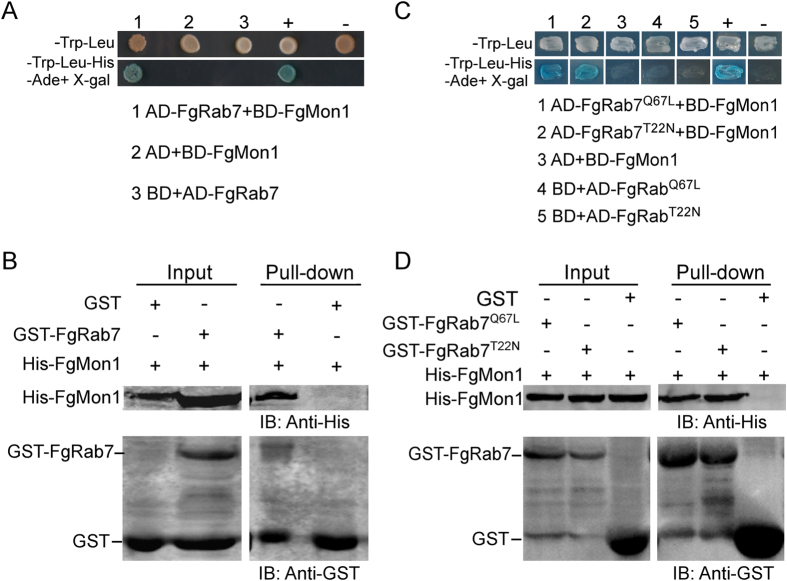
Assays for the interaction of FgMon1 and FgRab7, FgRab7^Q67L^, FgRab7^T22N^. (**A and C**) Yeast two hybrid assay. Yeast transformants expressing FgMon1 bait and the FgRab7 prey constructs were assayed for growth on SD-Trp-Leu and SD-Trp-Leu-His-Ade+X-gal plates. The interaction between pGBKT7-53 and pGADT7-T was used as the positive control (+), and non-interactions between pGBKT7-Lam and pGADT7-T (−), pGBKT7-FgMon1 and pGADT7, pGBKT7 and pGADT7-FgRab7^Q67L^, pGBKT7 and pGADT7-FgRab7^T22N^, pGADT7 and pGADT7-FgRab7 were used as negative controls. (B and D) *In vitro* pull down assay. Recombinant GST-FgRab7, GST-FgRab7^Q67L^, GST-FgRab7^T22N^ or GST- bound to glutathione Sepharose beads was incubated with *E. coli* cell lysate containing His-FgMon1. The eluted proteins was analyzed by immunoblot (IB) with monoclonal anti-His and monoclonal anti-GST antibodies.

To clarify the relationship between FgMon1 and FgRab7, yeast two hybrid (Y2H) and *in vitro* GST-pull down assays were carried out to test whether they interact with each other. The pGBKT7-FgMon1 bait and pGADT7-FgRab7 prey constructs were generated and co-transformed into yeast cell AH109. The result showed that FgRab7 interacts with FgMon1 in Y2H assay (Fig. 9A). This interaction was further confirmed by GST-pull down assay using GST-FgRab7 and His-FgMon1 fusion proteins (Fig. 9B), suggesting the direct association between FgRab7 and FgMon1. To further analyze the relationship between FgMon1 and FgRab7, we constructed GTP-associated version FgRab7^Q67L^ and GDP-associated version FgRab7^T22N^. Both Y2H and *in vitro* GST-pull down assays showed that FgMon1 interacts with FgRab7^Q67L^ and FgRab7^T22N^, respectively (Fig. 9C,D).

## Discussion

In *S. cerevisiae*, the Mon1-Ccz1 complex was found to function in cytoplasm to vacuole targeting (Cvt) pathway and autophagy pathway^7^. In addition, the complex also plays a role in endosomal membrane fusion machinery^8^. Recent studies reported that the Mon1-Ccz1 complex serve as the Rab7 GEF^25^. However, in our study only the *MON1* homolog but not *CCZ1* were found in *F. graminearum*, indicating other components might replace *CCZ1* or *CCZ1* might function redundantly in this pathogen. Besides, phylogenetic analysis showed Mon1 was well conserved in filamentous fungi. In addition, we generated the *FgMON1* gene deletion mutants of *F. graminearum*. The multiple defects of the *∆Fgmon1* mutant implicate that FgMon1 is a key protein for the development, infection and DON production of *F. graminearum*. Since DON is an important virulence factor in *F. graminearum*, in addition to its reduced growth rate, defects of the *∆Fgmon1* mutant in DON biosynthesis may also contribute to its defects in plant infection. In the rice blast fungus *M. oryzae*, MoMon1 is known to be involved in conidial morphology^28^. Similar to this, we found conidial morphology of the Δ*Fgmon1* mutant was also changed when compared with the wide type PH-1. Besides, the *∆Fgmon1* mutant was hypersensitive to cell wall perturbing agents, indicating FgMon1 might have a role in cell wall integrity maintenance.

Many genes involved in membrane fusion of the endomembrane system have been reported to have important roles in the development and pathogenicity of phytopathogens. Deletion of *MON1* in *M. oryzae* resulted in defects in vegetative growth, sporulation, autophagy, appressoria formation, pathogenicity and massive vacuole fragmentation^28^. SNARE proteins MoVam7 and MoSec22, play crucial roles in hyphal growth, conidiation, vacuole morphology, virulence and endocytosis^32^,^33^. Recent studies showed that FgVam7, FgYpt7, MoYpt7 plays roles similar to those of MoVam7, MoSec22 and MoMon1^13^,^23^,^24^, indicating that the proteins function in endomembrane system are important for the correct regulation of infection-related morphogenesis in different fungi.

In yeast, GFP-tagged Mon1 and Ccz1 mutants were found in punctate structures, which probably represent endosomes^7^,^25^,^34^, while in *Arabidopsis*, GFP-Mon1 mainly showed a cytosolic and endosomal localization^15^. Our data show that GFP-Mon1 localized in the cytosol and vacuoles in *F. graminearum*. This would be compatible with the recruitment of Mon1 to some membranes, as expected for a function in vacuolar fusion of Cvt vesicles and autophagosomes. In *M. oryzae* and *F. graminearum*, Rab7 proteins are thought to be localized to the vacuolar membrane, similar to that of in *S. cerevisiae* and *Arabidopsis*^15^,^23^,^24^,^35^,^36^. In yeast and animal cells, maturation of late endosomes from early endosomes require the conversion of Rab5-to-Rab7. Mon1-Ccz1 complex, the effectors of Rab5, could be recruited to the membrane by activated Rab5 protein and then bind Rab7. In addition, the Mon1-Ccz1 complex also influence Rab7 activation, and acts as an important link between Rab5 and Rab7^25^,^37^,^38^. Theoretically, wild-type Rab7 should contain a GDP-bound form that can be recruited by the Mon1-Ccz1 complex. However, the data in yeast and *Arabidopsis* revealed that the complex only interact with the GDP-locked version of Rab7^T22N^, which could due to the possible transient interaction between wild-type Rab7 and the Mon1-Ccz1 complex^15^,^34^. Our results showed that FgMon1 directly interacts with FgRab7 which showed similar phenotypes to that of FgMon1. In addition, we observed that FgMon1 specific interacts with GTP-associated version FgRab7^Q67L^ and GDP-associated version FgRab7^T22N^ in both Y2H and *in vitro* GST-pull down assays. This result is consistent with what have been found in *Caenorhabditis elegans*^37^. The Mon1:Ccz1 complex appears to facilitate the displacement of GDI (Rab Guanine Nucleotide Dissociation Inhibitors) from Rab7 and promote GTP loading of Rab7^37^. Wild-type FgRab7 should contain a GDP-bound form and GTP-bound form that can interact with FgMon1. Therefore, we speculate that FgMon1 may facilitates GTP loading of FgRab7 as well as GDI release, and a dynamic balance likely exist between FgMon1, FgRab7^Q67L^and FgRab7^T22N^, which might regulate the activity of Rab7. Besides, constitutively activated FgRab7 could rescue the defects of the Δ*Fgmon1* mutant, indicating that FgMon1 likely is a GEF of FgRab7, which is similar to that found in yeast. However, constitutively activated FgRab7 in the wide-type PH-1 caused no phenotypic changes, indicating the active form and the negative form of FgRab7 coexistence in the wide-type PH-1, and the transformation between both forms might be transient and in a dynamic balance.

In yeast cells several different transport pathways converge upon the vacuole and the Cvt process overlaps with macroautophagy, which non-selectively deliver cytosolic proteins and organelles to the vacuole for degradation and recycling^39^. Therefore, we speculated that fragmented vacuoles of the Δ*Fgmon1* mutant probably account for the delayed endocytosis, thereby influencing endosomal membrane fusion. In autophagy pathway, assembled autophagosome transported toward the vacuole and fuses with the membrane of vacuole to release the inner membrane structure and cargo^40^. We wonder whether the defect in vacuole morphology of Δ*Fgmon1* could influence the autophagy pathway or not. Autophagy is a process that cytoplasmic components and organelles of a cell are delivered to lysosomes for degradation. Under nutrient-deprived conditions, autophagy can be induced for cell survival. It is also a conserved mechanism from yeast to humans^41^,^42^. For example, autophagic dysfunction is associated with cancer, neurodegeneration, microbial infection and ageing^43^. In fact, many studies have been carried out using the N-terminal GFP-tagged Atg8 to monitor autophagy in yeast, mammals and filamentous fungi^40^,^44^. In our study, GFP-FgAtg8 cannot be delivered to fragmented vacuoles to degradation. In previous studies, Rab7 is also reported to be required for the fusion of autophagosome to the vacuole in yeast and other species^24^,^45^,^46^, and combined with our results, we concluded that the Δ*Fgmon1* mutant showed a defective in the fusion of autophagosomes and vacuoles which may also due to the fragmented vacuoles of the mutant. Fusion of autophagosomes with the vacuole and breakdown of the single-membrane autophagic body in the vacuole are critical steps in the autophagy pathway^24^. Therefore, we conclude that deletion of FgMon1 affects vacuole fusion, thus influence endocytosis and autophagy, and eventually affects the development and infection of the *F. graminearum*.

Taken together, we have identified and characterized *FgMON1*, a gene encoding a vacuolar fusion protein in *F. graminearum*, is important for hyphal growth, sexual reproduction, pathogenesis, vacuole fusion, endocytosis and autophagy. We also provide evidences that FgMon1 might act as a GEF of FgRab7 and directly interaction with FgRab7 in *F. graminearum*. However, relationships and interaction mechanisms between FgMon1 and FgRab7 need further studies.

## Methods

### Fungal strains and growth conditions

The wild type *F. graminearum* strain PH-1 and all other strains generated in this study were cultured on V8 juice agar plates at 25 °C. Cultures for genomic DNA and RNA isolation, conidiation in CMC medium and growth assays on CM, MM, 5xYEG media were performed as previously described^32^. Complete medium (CM) with SDS, CFW or monensin was used for stress response assays. For sexual reproduction, aerial hyphae of 10-day-old carrot agar cultures of the indicated strains were pressed down with 300 μl of sterile 0.1% Tween 20 as described^47^.

### Plant infection and DON production assays

For plant infection assays, conidia from 3-day-old CMC cultures were harvested and resuspended to 10^6^/ml or 10^5^/ml in sterile distilled water with 0.2% gelatin. Wheat germs were inoculated with 2 μl conidial suspensions (10^6^/ml) and examined at 10 dpi. Flowering wheat heads of cultivar Annong 8455 were drop-inoculated with 10 μl of conidium suspensions (10^5^/ml) at the sixth spikelet from the base of the spike. 10 μl of 0.2% gelatin served as controls. Symptomatic spikelets were examined and counted 14 dpi. For each treatment, 15 wheat heads were inoculated. For DON production assay, 50 g healthy wheat kernels was sterilized and inoculated with five mycelial plugs of each strain and incubation at 25 °C for 20 d. DON extraction and DON production quantification was performed as previously described^48^.

### qRT-PCR analysis

Total RNA samples were isolated from vegetative hyphae of PH-1 and *∆Fgmon1* mutant cultured in liquid YEPD for 2 days, and used for cDNA synthesis with the HiScript Q Select RT SuperMix for qPCR kit (Vazyme Biotech, Nanjing, China) following the instructions. The RT2 PCR Real-Time SYBR Green/ROX PCR master mix (TaKaRa, Dalian, China) was used for qRT-PCR analysis. Primer pairs TRI5QF/TRI5QR and TRI6QF/TRI6QR^13^ were used to amplify the *TRI5* and *TRI6* genes, respectively. The relative quantification of each transcript was calculated by the 2^-ΔΔCT^ method^49^ with the *F. graminearum* beta-tubulin gene *TUB2* as the internal control. For each gene, qRT-PCR assay repeated three times with three biological replicates.

### Generation of the GFP-*FgMON1*, GFP-*FgRAB7* and *FgRAB7* ^*Q67L*^ constructs

Fragment including the entire *FgMON1* or *FgRAB7* gene and its native promoter region, was amplified by PCR with primers from PH-1. The product was then cloned into pYF11 by the yeast gap repair approach^50^. The resulting plasmids were confirmed by sequencing analysis to contain the in-frame fusion constructs and transformed into the *∆Fgmon1* mutant, respectively. The resulting zeocin-resistant transformants were screened by PCR or confirmed by the presence of GFP signals. The primers are listed in Table S1.

### Yeast two hybrid and *in vitro* pull down assays

To examine the interaction between FgMon1 and FgRab7 using yeast two hybrid assays, the coding sequence of each tested gene was amplified from the cDNA of PH-1. The cDNA fragment of *FgMON1* was inserted into pGBKT7 as the bait construct, while the cDNA fragment of *FgRAB7*, *FgRAB7*^*Q67L*^and *FgRAB7*^*T22N*^ were cloned into pGADT7 as the prey construct. The pairs of the plasmids were co-transformed into the yeast strain AH109. In addition, a pair of plasmids, pGBKT7-53 and pGADT7-T, served as a positive control. The following pairs of plasmids were used as negative controls: pGBKT7-Lam and pGADT7-T; pGBKT7 and pGADT7-FgRab7; pGBKT7 and pGADT7-FgRab7^Q67L^; pGBKT7 and pGADT7-FgRab7^T22N^; pGADT7 and pGBKT7-FgMon1. Transformants were grown at 30 °C for 3 d on SD-Leu-Trp medium, and then transferred to the medium SD-Leu-Trp-Ade-His medium and containing 50 mM 3-aminotriazole (3-AT) to assess binding activity. The interaction was further examined by performing *β*-galactosidase activity using X-α-gal (80 μg/L). For the *in vitro* GST pull-down assay, the full-length cDNA of *FgRAB7*, *FgRAB7*^*Q67L*^, *FgRAB7*^*T22N*^ and *FgMON1* was inserted between the *EcoR*I and *Xho*I sites of vector pGEX-4T-2 and pET-32a, respectively. The resulting plasmids GST-FgRab7 and His-FgMon1 were separately introduced into the *E. coli* strain BL21. Soluble proteins were incubated with 30 μl glutathione agarose beads (Invitrogen) for 4 h at 4 °C. The beads were washed three times and then incubated with an equal amount of bacterial lysates containing His-FgMon1 for another 4 h at 4 °C. The beads were washed three times again, and the presence of His-FgMon1 was detected by immunoblot (IB) using anti-His antibody.

### Confocal microscopy and transmission electron microscopy assays

For endocytosis assay, hyphae were cultured in liquid YEPD medium for 12 h and stained by FM4-64 (N-3-triethylammoniumpropyl-4-p-diethylamino-phenyl-hexa-trienyl pyridinium dibromide) (Molecular Probes, USA) following the procedures described previously^51^. For vacuole staining, hyphae, conidia and germinated conidia were stained by CMAC (7-amino-4-chloromethylcoumarin) (Molecular Probes, USA) as described^52^. Photographs were taken under a confocal laser scanning microscopy. For autophagy assay, mycelium cultured in liquid CM medium for 10 h and then transferred to the nitrogen-limiting medium (MM-N) in the presence of 2 mM PMSF for 8 h. Transmission electron microscopy was carried out as previously described^32^.

## Additional Information

**How to cite this article**: Li, Y. *et al.* FgMon1, a guanine nucleotide exchange factor of FgRab7, is important for vacuole fusion, autophagy and plant infection in *Fusarium graminearum*. *Sci. Rep.* **5**, 18101; doi: 10.1038/srep18101 (2015).

## Supplementary Material

Supplementary Information

## Figures and Tables

**Table 1:** Phenotype analysis of the wild type, *∆Fgmon1* and *∆Fgrab7* mutants, *∆Fgmon1/FgRAB7* ^*Q67L*^ and WT/*FgRAB7* ^Q67L^ transformant in *F. graminearum*.

Strain	Colony Diameter (cm)^a^				Conidiation (x10^6^/ml)^b^	Conidial Length (μm)^c^	Conidial Morphology (%)^d^		DON^e^ (mg mg^-1^ergosterol)
	CM	V8	5xYEG	MM			≥3 Septa	≤2 Septa	
PH-1	7.3 ± 0.1	5.8 ± 0.1	5.3 ± 0.1	5.1± 0.1	1.23 ± 0.16	47.77 ± 4.94	91.2 ± 2.2	8.8 ± 2.2	4.10 ±0.10
*∆Fgmon1*	2.5 ± 0.1*	2.6 ± 0.1*	2.5 ± 0.1*	1.6 ± 0.1*	0.21 ± 0.04*	32.90 ± 3.96*	45.8 ± 3.1*	54.2 ± 3.1*	0.016 ±0.001*
*∆Fgrab7*	1.3 ± 0.1*	2.1 ± 0.1*	1.5 ± 0.1*	0.8 ± 0.1*	0.18 ± 0.03*	28.82 ± 5.40*	47.3 ± 2.8*	52.7 ± 2.8*	0.01±0.001*
*∆Fgmon1/FgMON1*	7.2 ± 0.1	5.8 ± 0.1	5.4 ± 0.1	5.1 ± 0.1	1.18 ± 0.11	47.10 ± 5.82	89.0 ± 4.5	11.0 ± 4.5	NA
*∆Fgrab7/FgRAB7*	6.7 ± 0.1	5.6 ± 0.1	5.1 ± 0.1	4.7 ± 0.1	1.14 ± 0.17	47.36 ± 4.76	88.7 ± 2.7	11.3 ± 2.7	NA
*∆Fgmon1/FgRAB7*^*Q67L*^	NA	5.2 ± 0.1	NA	NA	1.20 ± 0.09	47.11 ± 5.02	89.2 ± 3.2	10.8 ± 3.2	NA
WT/*FgRAB7*^Q67L^	NA	5.7 ± 0.1	NA	NA	1.21 ± 0.18	46.75 ± 5.36	88.3 ± 2.5	11.7 ± 2.5	NA

^a^Colony diameter of the indicated strains on different media after 3 days incubation at 25 °C.

^b^Quantification of the conidial production of the indicated strains form CMC cultures.

^c^Measurement of the conidial length of the indicated strains.

^d^Percentage of the abnormal conidia of the indicated strains.

^e^Percentage of DON production of each mutant in comparison with that of the wild-type. ±SD was calculated from three repeated experiments and asterisks indicate statistically significant differences (*p* < 0.01). NA, not assayed.

**Table 2:** Germination of the wild type, *∆Fgmon1* and *∆Fgrab7* mutants in YEPD medium.

Strain	Multi-polarity Germination (%)							
	4 h	6 h	8 h	10 h	12 h	14 h	16 h	18 h
PH-10	30.3 ± 2.5	48.3 ± 3.2	75.0 ± 3.0	89.3 ± 2.1	93.0 ± 2.0	95.0 ± 1.0	95.7 ± 0.5	97.0 ± 1.0
*∆Fgmon1*	11.0 ± 2.0*	16.3 ± 1.5*	23.7 ± 2.1*	45.3 ± 1.5*	65.3 ± 2.5*	75.7 ± 3.0*	86.3 ± 1.5*	91.0 ± 1.0
*∆Fgrab7*	10.3 ± 1.5*	20.7 ± 2.1*	49.0 ± 2.6*	83.0 ± 2.0	91.7 ± 1.5	NA	NA	NA
*∆Fgmon1/FgMON1*	29.7 ± 3.1	47.7 ± 2.5	73.0 ± 3.0	88.7 ± 2.5	92.0 ± 2.6	94.0 ± 1.0	94.7 ± 1.5	95.3 ± 1.5
*∆Fgrab7/FgRAB7*	30.0 ± 2.0	48.0 ± 2.0	72.7 ± 3.1	87.0 ± 1.7	93.7 ± 1.5	NA	NA	NA

±SD was calculated from three repeated experiments and asterisks indicate statistically significant differences (*p* < 0.01). NA, not assayed.
