# Supplementary material for: FgMon1, a guanine nucleotide exchange factor of FgRab7, is important for vacuole fusion, autophagy and plant infection in Fusarium graminearum
Source: Sci Rep. 2015 Dec 10;5:18101. doi: 10.1038/srep18101 (PMC4674805; doi:10.1038/srep18101)
Supplement: Supplementary Information [file srep18101-s1.doc]

**FgMon1, a guanine nucleotide exchange factor of FgRab7, is important for vacuole fusion, autophagy and plant infection in *Fusarium graminearum***

Ying Li^1^, Bing Li^1^, Luping Liu^1^, Huaigu Chen^2^, Haifeng Zhang^1*^, Xiaobo Zheng^1^, and Zhengguang Zhang^1^

^1^Department of Plant Pathology, College of Plant Protection, Nanjing Agricultural University, and Key Laboratory of Integrated Management of Crop Diseases and Pests, Ministry of Education, Nanjing 210095, China

^2^ Institute of Plant Protection, Jiangsu Academy of Agricultural Sciences, Nanjing 210014, China

*Corresponding author: Haifeng Zhang

Tel: 86-25-84396436

Email: hfzhang@njau.edu.cn

**Figure S1**


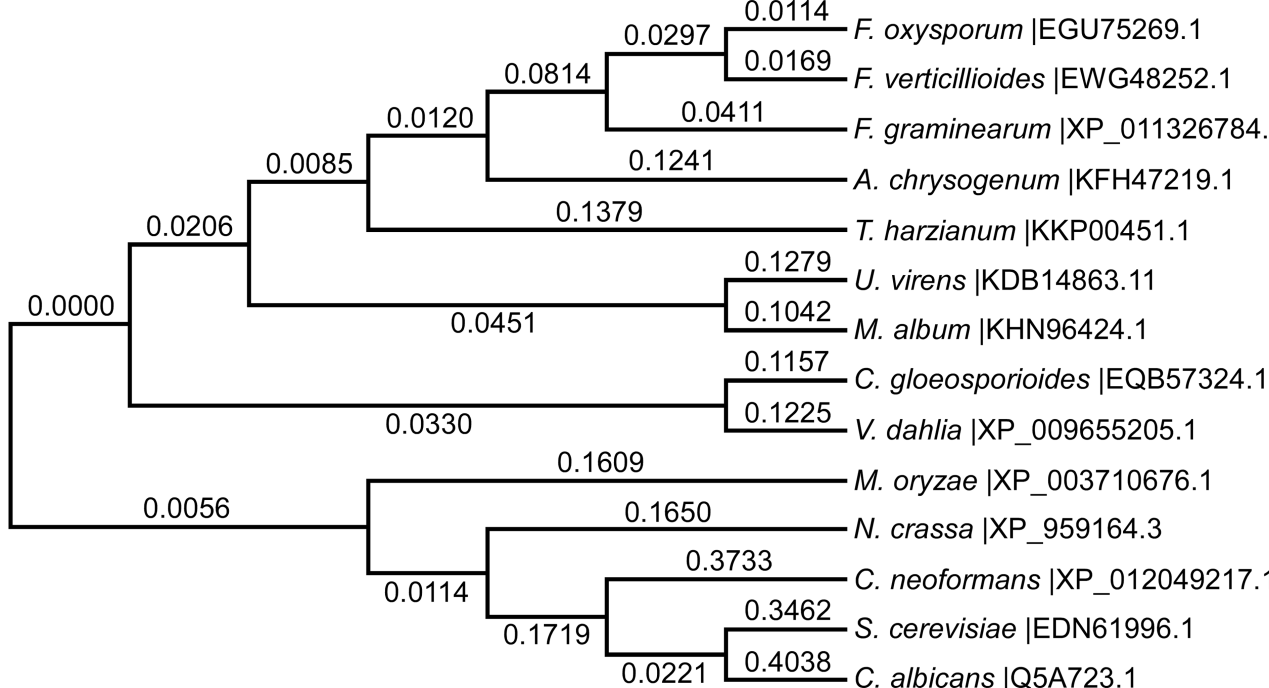


**Figure S1. Phylogenetic analysis of FgMon1 and its homologs from other fungi.** Including *Fusarium oxysporum*, *Fusarium verticillioides*, *Fusarium graminearum*, *Acremonium chrysogenum*, *Trichoderma harzianum*, *Ustilaginoidea virens*, *Metarhizium album*, *Colletotrichum gloeosporioides*, *Verticillium dahlia*, *Magnaporthe oryzae*, *Neurospora crassa*, *Cryptococcus neoformans*, *Saccharomyces cerevisiae*, and *Candida albicans*.

**Figure S2**


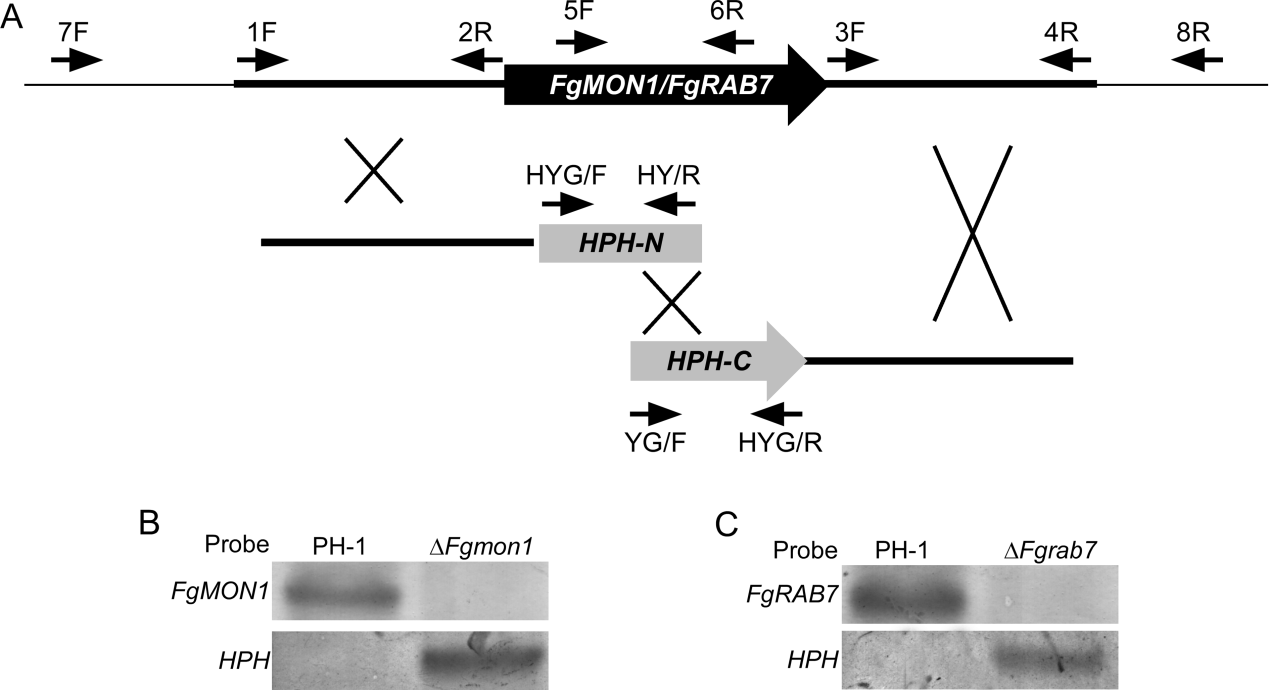


**Figure S2. Generation of the *FgMON1* and *FgRAB7* deletion mutants.** (A) Schematic diagram of the *FgMON1* and *FgRAB7* gene, and gene replacement construct. (B) Southern blot analysis of *Xho* I or *Sac* I-digested genomic DNA of the wild type PH-1 and ∆*Fgmon1* or ∆*Fgrab7* mutant hybridized with the gene and *HPH* probes, respectively.

**Figure S3**

**
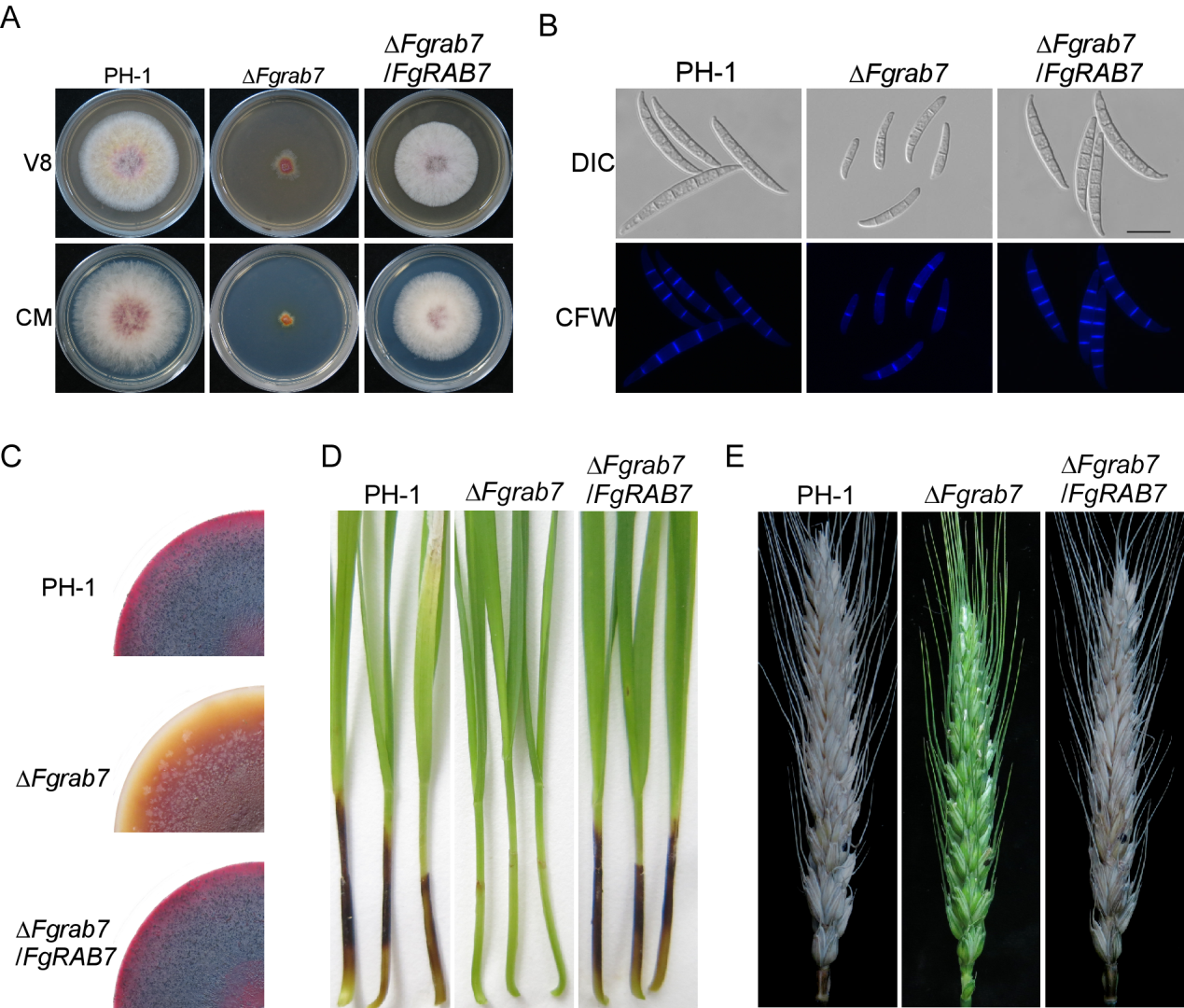
**

**Figure S3. Phenotype defects of the ∆*Fgrab7* mutant.** (A) Three-day-old cultures of the wild type PH-1, ∆*Fgrab7* mutant and the complemented transformant ∆*Fgrab7*/*FgRAB7* on V8 and CM plates. (B) Conidial morphology of the indicated strains. (C) Self-crossing plates of the indicated strains at 10 days post-fertilization. (D) Wheat germ infection assay. The infections were examined at 10 days post inoculation (dpi). (E) Flowering wheat heads infection assay. Photographs were taken at 14 dpi.

**Figure S4**


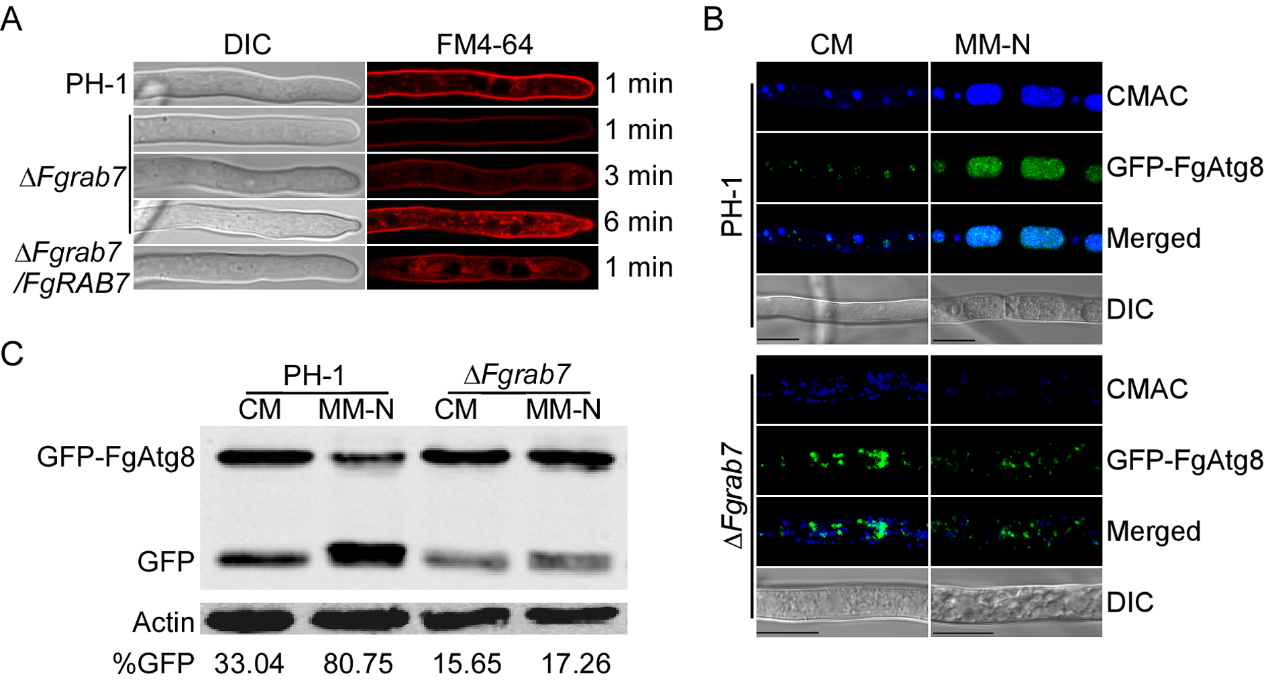


**Figure S4. Assays for the defects of the ∆*Fgrab7* mutant in endocytosis and autophagy.** (A) Hyphae of PH-1, ∆*Fgrab7* mutant and ∆*Fgrab7*/*FgRAB7* were stained with FM4-64 and examined by DIC or epifluorescence microscopy. (B) PH-1 and ∆*Fgrab7* mutant expressing GFP-FgAtg8 were grown in liquid CM medium at 25°C for 10 h, and shifted to liquid MM-N medium with 2 mM PMSF for 8 h. Mycelia were stained with CMAC and examined by DIC or epifluorescence microscopy. Scale bar = 10 μm. (C) GFP-FgAtg8 proteolysis assays of PH-1 and ∆*Fgrab7* mutant.

**Table S1. Primers used in this study.**

| Primer | Sequence (5’-3’) | Application |
| --- | --- | --- |
| FgMon1-1F | CGAGCTTGTAAAGCCTTGGATC | Amplify *FgMON1* 5’ flank sequence, for gene knock out |
| FgMon1-2R | TTGACCTCCACTAGCTCCAGCCAAGCCTGCACCCAGAATCGTACGACCT |
| FgMon1-3F | CAAAGGAATAGAGTAGATGCCGACCGTATCGAGGTCTGGTTTCCTCTT | Amplify *FgMON1* 3’ flank sequence, for gene knock out |
| FgMon1-4R | CTGTCTGCTTGGATAGTTCACA |
| FgMon1-5F | TACTTGATCCTATCATCGGCCG | Amplify *FgMON1* gene probe, for southern blot and transformants screen |
| FgMon1-6R | TTCGAAGTCGAAGACACTCCAG |
| FgMon1-7F | AAGTCGTTTCGCGACAGTCG | Transformants screen |
| FgMon1-8R | TGGTCAACGGGTCTCTGGAT |
| HYG/F | GGCTTGGCTGGAGCTAGTGGAGGTCAA | Amplify *HPH-N* sequence |
| HY/R | GTATTGACCGATTCCTTGCGGTCCGAA |
| YG/F | GATGTAGGAGGGCGTGGATATGTCCT | Amplify *HPH-C* sequence |
| HYG/R | CGGTCGGCATCTACTCTATTCCTTTG |
| FgRab7-1F | CGTTCGTGTAAAGATGCGAC | Amplify *FgRAB7* 5’ flank sequence, for gene knock out |
| FgRab7-2R | TTGACCTCCACTAGCTCCAGCCAAGCC AGCGGTGCTCAATGACGTTATC |
| FgRab7-3F | CAAAGGAATAGAGTAGATGCCGACCG AGTGGATAGACGAATAGCGG | Amplify *FgRAB7* 3’ flank sequence, for gene knock out |
| FgRab7-4R | ACAAACTTGGCGACGAGGTCAA |
| FgRab7-5F | GGTGTTGGAAAGACCAGCTT | Amplify *FgRAB7* gene probe for southern blot and transformants screen |
| FgRab7-6R | ATCAGTATACATGCTCCGGTCG |
| FgRab7-7F | GTCTGCATCGTTACCAGCATCA | Transformants screen |
| FgRab7-8R | CCTTCTCCTTCAGATCCTCA |
| FgMon1-NPF | ACTCACTATAGGGCGAATTGGGTACTCAAATTGGTTACGGCTCTGACTGGTTATCGAA | *FgMON1* complementation, native promoter |
| FgMon1-NPR | CACCACCCCGGTGAACAGCTCCTCGCCCTTGCTCACGAACACACCGCCTCCAATAA |
| FgRab7-NPF | ACTCACTATAGGGCGAATTGGGTACTCAAATTGGTTCTGGATTCGGTGCAAGTAAACC | *FgRAB7* complementation, native promoter |
| FgRab7-NPR | CACCACCCCGGTGAACAGCTCCTCGCCCTTGCTCACACAAGCACAGCCATCGCGGTCGTT |
| FgMon1-RP1F | TTT CGT AGG AAC CCA ATC TTC AAA ATGGTGAGCAAGGGCGAGGA | *FgMON1* complementation, constitutive promoter |
| FgMon1-RP 2R | GGTTTATACTGTCAGAGTCCAT CTTGTACAGCTCGTCCATGC |
| FgMon1-RP3F | ATGGACTCTGACAGTATAAACC |
| FgMon1-RP4R | GTGGCGGATCTTGAAGTTCA TCAGAACACACCGCCTCCAAT |
| FgMon1-32a-1F | CGAGAATTCATGGACTCTGACAGTATAAACC | *FgMON1* prokaryotic expression construct, for GST pull down |
| FgMon1-32a-2R | CGTCTCGAG TCAGAACACACCGCCTCCAAT |
| FgRab7-4T-2-1F | CGCGTG GATCCCCAGG AATTCAGATGTCTTCTCGAAAGAAGGTTC | *FgRAB7* prokaryotic expression construct, for GST pull down |
| FgRab7-4T-2-2R | GCGATGGCTGTGCTTGTTAA CTCGAGCGGCCGCATC GTGA |
| FgRab7 ^Q67L^ -4T-2-1F | CGCGTGGATCCCCAGGAATTCAGATGTCTTCTCGAAAGAAGGTTC | *FgRAB7* *^Q67L^* prokaryotic expression construct, for GST pull down |
| FgRab7 ^Q67L^ -4T-2-2R | GCGATGGCTGTGCTTGTTAACTCGAGCGGCCGCATC GTGA |
| FgRab7 ^T22N^ -4T-2-1F | CGCGTGGATCCCCAGGAATTCAGATGTCTTCTCGAAAGAAGGTTC | *FgRAB7* *^Q67L^* prokaryotic expression construct, for GST pull down |
| FgRab7 ^T22N^ -4T-2-2R | GCGATGGCTGTGCTTGTTAACTCGAGCGGCCGCATC GTGA |
| FgMon1-BD-1F | CATATGATGGACTCTGACAGTATAAACC | *FgMON1* yeast expression construct, for yeast two hybrid |
| FgMon-BD-2R | GAATTCTCAGAACACACCGCCTCCAATA |
| FgRab7-AD-1F | GACCAT ATG ATGTCTTCTCGAAAGAAGGTTC | *FgRAB7* yeast expression construct, for yeast two hybrid |
| FgRab7-AD-2R | GACGAA TTC TTAACAAGCACAGCCATCGC |
| FgRab7^Q67L^- AD-1F | GTACCAGATTACGCTCATATGATGTCTTCTCGAAAGAAGGTTC | *FgRAB7^Q67L^* yeast expression construct, for yeast two hybrid |
| FgRab7^Q67L^- AD-2R | GGAATCGTTCTAGACCGGCAGT |
| FgRab7^Q67L^- AD-3F | ACTGCCGGTCTAGAACGATTCC |
| FgRab7^Q67L^- AD-4R | ATGCCCACCCGGGTGGAATTCTTAACAAGCACAGCCATCGC |
| FgRab7^T22N^- AD-1F | GTACCAGATTACGCTCATATGATGTCTTCTCGAAAGAAGGTTC | *FgRAB7^T22N^* yeast expression construct, for yeast two hybrid |
| FgRab7^T22N^- AD-2R | CATCAAGCTGTTCTTTCCAAC |
| FgRab7^T22N^- AD-3F | GTTGGAAAGAACAGCTTGATG |
| FgRab7^T22N^- AD-4R | ATGCCCACCCGGGTGGAATTCTTAACAAGCACAGCCATCGC |
| FgRab-Q67L-1F | TTT CGT AGG AAC CCA ATC TTC AAA ATGTCTTCTCGAAAGAAG | For constitutively activate *FgRAB7* construct |
| FgRab-Q67L-2R | GGAATCGTTCTAGACCGGCAGT |
| FgRab-Q67L-3F | A CTGCCGGTCTAGAACGATTCC |
| FgRab-Q67L-4R | CACCACCCCGGTGAACAGCTCCTCGCCCTTGCTCACTTAACAAGCACAGCCATCGC |
| TRI5-QF | TGAGGGATGTTGGATTGAGCA | *FgTRI5* qRT-PCR analysis |
| TRI5-QR | TGCTTCCGCTCATCAAACAGG |
| TRI6-QF | GCTACTCAGAATGCCCTCAG | *FgTRI6* qRT-PCR analysis |
| TRI6-QR | CGCATGTTATCCACCCTGCTA |
| Tub-F | GTCAGTGCGGTAACCAAATCG | Reference gene for qRT-PCR analysis |
| Tub-R | CTCAGAGGTGCCGTTGTAAAC |
| FgAtg8-1F | TTTCGTAGGAACCAATCTTCAAAATGGTGAGCAAGGGCGAGGAG | *For GFP-FgATG8* fusion construct, constitutive promoter |
| FgAtg8-2R | CTTGTACAGCTCGTCCATGCCGAGAGTGAT |
| FgAtg8-3F | GCATGGACGAGCTGTACAAGATGCGCAGCAAATTCAAGGACG |
| FgAtg8-4R | CTTCTCGTTGGGGTCTTTGCTCAGGTTACGCTTCGCCAAAAGTGTT |
| FgRab7CAqRT-F | GAGTTTCTCATCCAGGCTTCTC | For constitutive transformant qRT-PCR analysis |
| FgRab7CAqRT-R | GGCTCGCTTGTTGGAAATAAC |
